# Machine Learning for Brain Images Classification of Two Language Speakers

**DOI:** 10.1155/2020/9045456

**Published:** 2020-06-06

**Authors:** Alejandro-Israel Barranco-Gutiérrez

**Affiliations:** Cátedras CONACyT—TecNM Celaya, Celaya 38010, Mexico

## Abstract

The image analysis of the brain with machine learning continues to be a relevant work for the detection of different characteristics of this complex organ. Recent research has observed that there are differences in the structure of the brain, specifically in white matter, when learning and using a second language. This work focuses on knowing the brain from the classification of Magnetic Resonance Images (MRIs) of bilingual and monolingual people who have English as their common language. Different artificial neural networks of a hidden layer were tested until reaching two neurons in that layer. The number of entries used was nine hundred and the classifier registered a high percentage of effectiveness. The training was supervised which could be improved in a future investigation. This task is usually carried out by an expert human with Tract-Based Spatial Statistics analysis and fractional anisotropy expressed in different colors on a screen. So, this proposal presents another option to quantitatively analyse this type of phenomena which allows to contribute to neuroscience by automatically detecting bilingual people of monolinguals by using machine learning from MRIs. This reinforces what is reported in manual detections and the way that a machine can do it.

## 1. Introduction

The use of Magnetic Resonance Imaging (MRI) has reached a high degree of sophistication because it is useful for analysing the brain and detecting its diseases; however, the structure quantification and tissues has not yet been completely solved [1, 2]. Several machine learning (ML) techniques have been implemented to classify brain activity, diseases, and behaviours and have achieved approximate solutions from this discipline [3–6]. Even due to the remarkable results of these techniques, dedicated hardware is currently being created for ML tasks [7–9]. An interesting example of ML applied to MRIs is presented by [10]; in their study, they investigated deep learning framework algorithms for predicting the Soil Organic Matter (SOM) content by VIS-NIR spectroscopy. Based on fractional-order derivative (1.5) spectral variation, they compared Backpropagation Neural Network (BPN), Multilayer Perceptron (MLP), and Convolutional Neural Network (CNN), including LeNet5 and DenseNet10 with full-spectrum data (203 variables) and a subset of 67 variables highly correlated with the SOM content (r2 values > 0.4). Their results indicate that deep learning methods including the MLP and CNN can be used to predict the SOM content from VIS-NIR soil spectra, each displaying state-of-the-art performance. In [11], a DL model based on a CNN is proposed to classify different brain tumor types using two publicly available datasets. The former one classifies tumors into (meningioma, glioma, and pituitary tumor). The other one differentiates between the three glioma grades (Grade II, Grade III, and Grade IV). The datasets include 233 and 73 patients with a total of 3064 and 516 images on T1-weighted contrast-enhanced images for the first and second datasets, respectively. The proposed network structure achieves a significant performance. The results indicate the ability of the model for brain tumor multiclassification purposes. The authors of [12] propose a novel framework for the classification of fetal brain at an early age (before the fetus is born). This is a study to classify fetuses' brain abnormalities of widespread Gestational Ages (GAs). The study incorporates several machine learning classifiers, such as Diagonal Quadratic Discriminates Analysis (DQDA), K-Nearest Neighbour (K-NN), random forest, naïve Bayes, and Radial Basis Function (RBF) neural network classifiers. Moreover, several bagging and Adaboosting ensemble models have been constructed using random forest, naïve Bayes, and RBF network classifiers. The performances of these ensembles have been compared with their individual models. Theirs results show that the approach can successfully identify and classify numerous types of defects within MRI images of the fetal brain of various GAs. Using the KNN classifier, it is able to achieve the highest classification accuracy. In [13], the authors present an intuitionistic fuzzy kernel clustering (IIFKC) method using intuitionistic fuzzy set theory that encompasses a kernel-based distance function. The proposed method preserves the image information, insensitive to noise, and free of prerequisites of fine-tuning parameters. The segmentation results attained on the real and simulated MRI brain image exhibits the efficiency of the IIFKC method and enhances the performance in comparison with the existing methods in terms of similarity index, Jaccard coefficients, and execution time. Varuna-Shree and Kumar [14] present a noise removal technique, extraction of Gray-Level Co-occurrence Matrix (GLCM) features, and Discrete Wavelet Transformation- (DWT-) based brain tumor region growing segmentation to reduce the complexity and improve the performance. This was followed by morphological filtering which removes the noise that can be formed after segmentation. The probabilistic neural network classifier was used to train and test the performance accuracy in the detection of tumor location in brain MRI images. In [15], a study of Berkeley Wavelet Transformation- (BWT-) based brain tumor segmentation is shown. Furthermore, to improve the accuracy and quality rate of the Support Vector Machine- (SVM-) based classifier, relevant features are extracted from each segmented tissue. The experimental results of proposed technique have been evaluated and validated for performance and quality analysis on magnetic resonance brain images, based on accuracy, sensitivity, specificity, and dice similarity index coefficient. The experimental effectiveness of the proposed technique for identifying normal and abnormal tissues from brain MRIs. The research reported in [16] proposes a Probabilistic Neural Network-Radial Basis Function method to increase the classification accuracy in tumor functional brain images. The classification is carried out by extracting the features by using multilevel wavelet method. Then, the morphological filtering technique is used in segmentation process where the Region of Interest (ROI) areas of the brain functional images are compared with the neighbourhood pixels. This technique yields a high accuracy for the functional brain images. This procedure is efficient to diagnose the tumor region in early stage itself.

On the contrary, current research studies indicate that learning and using a Second Language (L2) can affect the structure of the brain, White Matter (WM) tracts, and Gray Matter (GM) tracts [17–19]. This observation comes from research studies that analyse early and older bilingual people who have been using their first and second languages for several years [20–22]. This change caused by L2 is presumed positive because lifelong bilingualism contributes to the cognitive reserve against decreasing the integrity of white matter in aging [23, 24]. Sulpizio et al. affirm that “*currently no agreement on factor modulates most effectively and enduringly brain plasticity in bilingual individuals*” [25]. They consider that the classification of bilingualism versus monolingualism can hide detailed changes in neurons, derived from experience in language practice, thus leading to variable and often conflicting findings. And they present the effect of the Age of Acquisition (AoA). These findings shed new light on the importance of modelling bilingualism as a gradient measure rather than an all-or-none phenomenon.

In this work, we analysed the literature regarding the classification of brain images with machine learning techniques from magnetic resonance imaging. This is to review the techniques that have been used for classification and to observe the importance of the sensor to measure the variable that needs to be analysed as well as the preprocessing necessary to feed the classification system. This is in order to help the classifier in his task. In this case, it has the hypothesis that you can classify brain images by examining the corpus callosum bilaterally, including the genu, the body, and the previous part of the splenium from MRIs. In order to prove it, the work was organized as follows. In Section 2, the characteristics of the database as well as the process to obtain the variation of data that feeds information to the neural network are explained.  Section 3 presents the experimental outcomes of the classification using different architectures of neural networks.  Section 4 presents the author's interpretation of the results. Finally, the conclusions and future works are presented.

## 2. Materials and Methods

### 2.1. Database

**Figure 1:**
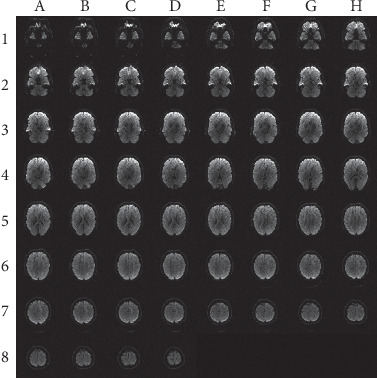
Set of 60 images of cross sections of the brain of the volunteer 101.

The raw material for this work is the L2struc database hosted in XNAT Central, freely available online through the PNAS open access option. The share data was approved by the University of Reading Research Ethics Committee. All participants provided written informed consent prior to participating [17]. It was used a 3.0-Tesla Siemens MAGNETOM Trio MRI scanner with Syngo software and 36-channel Head Matrix coil to acquire a whole-brain diffusion-weighted Echo-Plannar Imaging image (two averages, 30 directions, 60 axial slices; slice thickness, 2 mm, no interslice gap; field of view, 256 × 256 mm; acquisition matrix, 128 × 128; voxel size, 2 mm isotropic; echo time, 93 ms; repetition time, 8,200 ms; *b*-value, 1,000 s/mm^2^). A 3D brain shot participant number 101 is presented in Figure 1. A set of 60 cross sections can be observed at different heights of the brain, arranged in rows from 1 to 8 and in columns from A to H. For the implementation of the method, *Matlab 2019* and its *“dicomread”* function were used to manipulate the RMI files while the implementation of the Artificial Neural Network (ANN) was carried out with *Matlab's* “*nprtool*” tool.

### 2.2. Data Variance

**Figure 2:**
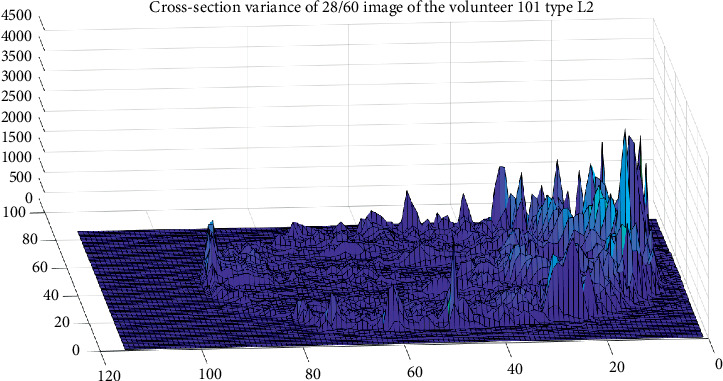
Set of 62 cross-section variance of brain images for the volunteer type L2.

**Figure 3:**
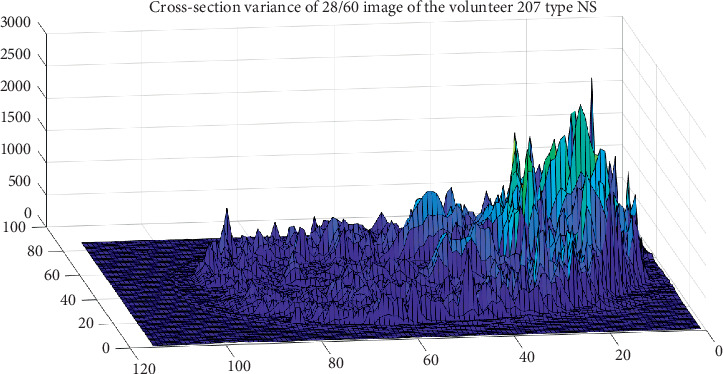
Set of 62 cross-section variance of brain images for the volunteer type NS.

The cross-section variance for each voxel was performed to locate the areas where major changes exist in the same participant on different tomography. As an example, in Figure 2, it has the level variance of 62 tomography of the same brain slice, in order to observe what are the interest areas regardless of whether they speak one or two languages. It can see more changes in the frontal and occipital lobes. And the same happens with the variances presented in Figure 3.

The same procedure was performed for a Native Speaker (NS) participant. The result is shown in Figure 3, where it can be seen that the corpus callosum has higher variance levels in NS participant than L2 participant. Both NS and L2 participants are male with PhD studies.

**Figure 4:**
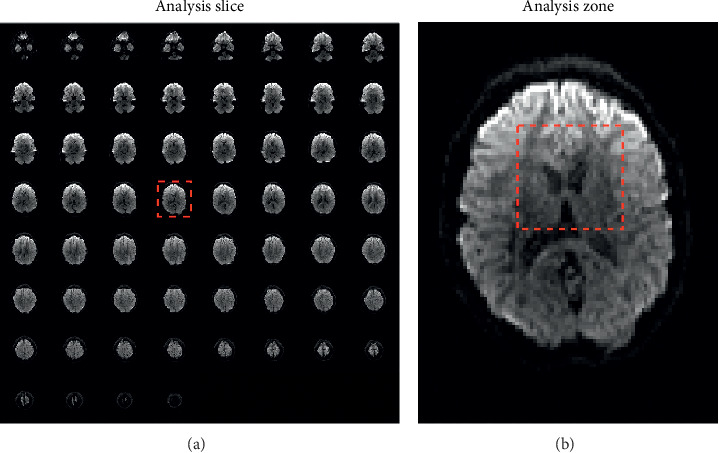
Selected data for the classification of brain images of only English speakers (NS) or two languages (L2). (a) Analysis slice and (b) slice zone.

The literature indicates that the differences between bilingual and monolingual are reflected in the corpus callosum bilaterally, this is why that brain zone was selected to classify between volunteers L2 and NS. Also, the 30 by 30 matrix form is to facilitate data acquisition, which could be further optimized.  Figure 4 shows the analysis slice (D4 respect to Figure 1) among the 60, existing for each tomography, of the 62 performed for each volunteer, according to Figures 2 and 3, together with the analysis performed in [17].

The 75% of the data were taken for training, 10% for validation, and 15% for testing. Due to size defects in the image of participant 105, it was removed from the experiment. Therefore, the tomography of nineteen L2-type participants and twenty-five NS-type participants was used. To reinforce the training, the training dataset was repeated three times and this significantly reduced the error. The 30 by 30 matrix was reshaped in an input vector of 900 elements where each element was the normalized mean of 62 voxels of each participant. Normalization was carried out with respect to the maximum of each participant:

(1) $$\begin{array}{c} {\bar{x}}_{ij}=\frac{x_{ij}}{\max\left(x_{ij}^{t}\right)}, \end{array}$$

where *i* and *j* are the row and column indices, respectively, and *t* is the tomography index.

### 2.3. Machine Learning

**Figure 5:**
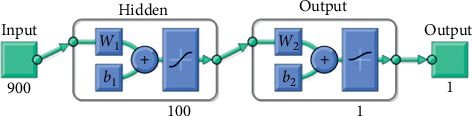
Architecture of the initial artificial neural network used to classify RMIs of L2 and NS participants.

In order to measure the ease or complexity with which a machine learning system can classify between L2 and NS speakers MRIs, different neural network architectures were tested until it reached a simple perceptron, and one of them is shown in Figure 5. The tests were carried out using single hidden layer neural networks, varying its number of neurons. The feedforward architecture of the neural networks used is shown as follows:

(2) $$\begin{array}{c} \text{Output}=\log\text{sig}\left(W_{2}*\;\tan\text{sig}\left(I*W_{1}+b_{1}\right)+b_{2}\right), \end{array}$$

where *I* is the inputs vector, *W*_1_ is the weights vector from inputs to the hidden layer, *b*_1_ the first layer bias vector, *W*_2_ is the weights vector from the hidden layer to the output, *b*_2_ the output layer bias vector, tansig is the hyperbolic tangent sigmoid transfer function, and logsig is the log-sigmoid transfer function.

The experiment used the nprtool, and this data classification library only needs the input data, the targets in the output, and the number of neurons in the hidden layer to train a neural network. The pattern recognition network in MATLAB uses the default Scaled Conjugate Gradient Backpropagation algorithm for training. The data were randomly divided into 92, 20, and 20 samples for training, validation, and testing, respectively.

## 3. Results

In order to measure the efficiency in the L2 and NS participant detection using different quantity of neurons in the hidden layer, three representative tests were realized and reported in this section. Therefore, the error vs. epochs used for the training, validation, and test performances were plotted. And also, their Receiver Operating Characteristic (ROC) curves for training, validation, testing, and three together were constructed, which serve to evaluate the classifiers performance. In the ROC curves, it is possible to see which of the curves is nearby to the maximum value of the vertical axis (True Positive Rate) and similarly for the horizontal axis (False Positive Rate). Mathematically it can be said that the classifier with more area under the ROC curve has the best efficiency.

**Figure 6:**
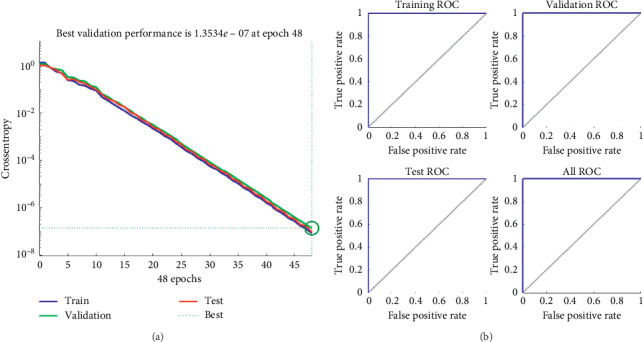
(a) The error vs. epochs used for the training, validation, and test performances. (b) ROC (Receiver Operating Characteristic) training, validation, and testing curves using one hundred hidden neurons.

The first experiment used one hundred neurons, its error for each epoch is shown in Figure 6(a), and its ROC curves in Figure 6(b).

**Figure 7:**
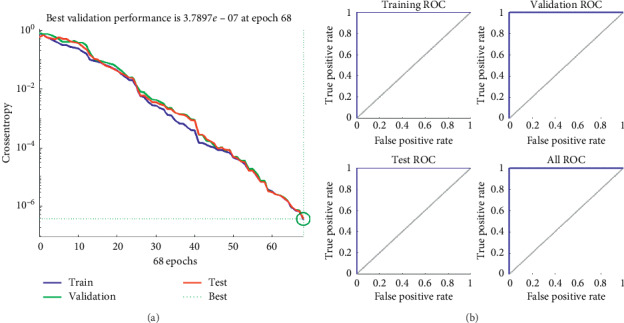
(a) The error vs. epochs used for the training, validation, and test performances. (b) ROC (Receiver Operating Characteristic) training, validation, and testing curves using two hidden neurons.

In the same way, the second experiment used one two neurons, and its error for each epoch is shown in Figure 7(a), and its ROC curves in Figure 7(b).

**Figure 8:**
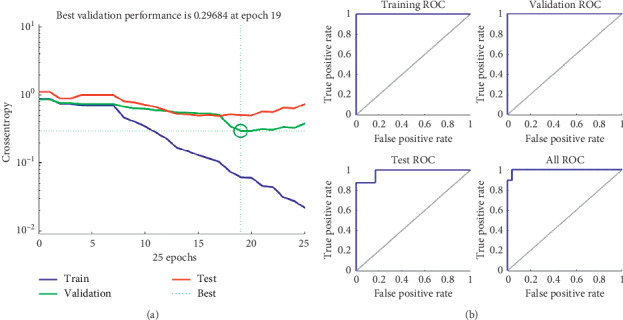
(a) The error vs. epochs used for the training, validation, and test performances. (b) ROC (Receiver Operating Characteristic) training, validation, and testing curves using one hidden neuron.

The last experiment used one neuron, and its error for each epoch is shown in Figure 8(a), and its ROC curves in Figure 8(b).

## 4. Discussion

According to the results, it can be confirmed from machine learning what recent literature asserts regarding structural changes in the brain was induced by speaking two languages. In this work, it shows that also an ANN finds differences in WM from bilingual people who learn their second language (L2) and are active users of both languages. Lately, this RMI analysis is carried out with Tract-Based Spatial Statistics, which represents a dependence on the talent of the statistical analyst. In contrast to artificial neural networks, the analysis can be automated, which is demonstrated and contributed in this work, and is relatively an easy task for a machine learning system.

With only the central section of the brain, it was possible to classify between L2 and NS participants with a high degree of efficiency, as shown by the ROC curves of Figures 6 and 7. This is very important to observe how machine learning tools can help in the analysis of brain image, in this case of the RMI type. And even more, it was observed that when using the aforementioned RMI scanner, the analysis of spatial statistics based on the tract and fractional anisotropy requires 128 × 128 × 0 inputs, while the red neural uses 30 × 30 × 1 inputs to decide if a participant is of type L2 or type NS, which represents a substantial reduction in information processing. Regarding the computational complexity of the forward neural network, it is of type *O* (*n*), where *n* is the number of inputs and the training method is the classic backpropagation, and this is linear in the number of training samples, that is, the operation to update the weights for a single sample is constant.

## 5. Conclusions

The study of the brain continues to be very active due to its complexity. Although there is a broad vision of its role within the body, there are still many unknowns to solve. To know more about it, this work developed a machine learning system for brain image classification of two language speakers. This is very relevant because it opens another option to quantitatively know the brain from machine learning, since the tool used par excellence for this type of analysis is the Tract-Based Spatial Statistics.

The classification was applied to magnetic resonance imaging of a group of 19 people who speak English as a second language and who are 13 to 374 months using it, with an average of 10.5 years of age in the English language acquisition, and also, from a second group of 25 participants who only speak one language and their native language is English. For this task, different artificial neural networks of a hidden layer with 900 inputs were designed. It was experimented with different amounts of hidden neurons until reaching a neuron and obtaining good results, although it was chosen to use two neurons to ensure 100% effectiveness in the classification of images from the database used.

The system can be extended with an investigation that reduces the number of ANN entries by analysing the voxels that provide more and better information for classification. The way to describe language learning from the brain structure in a gradual and quantitative manner could also be sought.

## Data Availability

The RMIs reported in this paper have been downloaded from XNAT Central, https://central.xnat.org (Project ID code L2struc). The participants' information is at http://www.pnas.org/lookup/suppl/doi:10.1073/pnas.1414183112/-/DCSupplemental.
